# *Nr5a2 *promotes cancer stem cell properties and tumorigenesis in nonsmall cell lung cancer by regulating *Nanog*

**DOI:** 10.1002/cam4.1992

**Published:** 2019-02-10

**Authors:** Ting Ye, Jingyuan Li, Zhiwei Sun, Yongli Liu, Liangsheng Kong, Shixia Zhou, Junlin Tang, Jianyu Wang, H. Rosie Xing

**Affiliations:** ^1^ Laboratory of Translational Cancer Stem Cell Research Institute of Life Sciences, Chongqing Medical University Chongqing China; ^2^ College of Biomedical Engineering State Key Laboratory of Ultrasound Engineering in Medicine, Chongqing Medical University Chongqing China

**Keywords:** cancer stem cell, *Nanog*, non‐small cell lung cancer, *Nr5a2*, tumorigenesis

## Abstract

Lung cancer has the highest mortality rate due to late diagnosis and high incidence of metastasis. Cancer stem cells (CSCs) are a subgroup of cancer cells with self‐renewal capability similar to that of normal stem cells (NSCs). While CSCs may play an important role in cancer progression, mechanisms underlying CSC self‐renewal and the relationship between self‐renewal of the NSCs and CSCs remain elusive. The orphan nuclear receptor *Nr5a2* is a transcriptional factor, and a regulator of stemness of embryonic stem cells and induced pluripotent stem cells. However, whether *Nr5a2* regulates the self‐renewal of lung CSCs is unknown. Here, we showed the diagnostic and prognostic values of elevated *Nr5a2* expression in human lung cancer. We generated the mouse LLC‐SD lung carcinoma CSC cellular model in which *Nr5a2* expression was enhanced. Using the LLC‐SD model, through transient and stable siRNA interference of *Nr5a2* expression, we provided convincing evidence for a regulatory role of *Nr5a2* in the maintenance of lung CSC self‐renewal and stem cell properties in vitro. Further, using the syngeneic and orthotopic lung transplantation model, we elucidated augmented cancer biological properties associated with *Nr5a2* promotion of LLC‐SD self‐renewal. More importantly, we revealed that *Nr5a2*’s regulatory role in promoting LLC‐SD self‐renewal is mediated by transcriptional activation of its direct target *Nanog*. Taken together, in this study, we have provided convincing evidence in vitro and in vivo demonstrating that *Nr5a2* can induce lung CSC properties and promote tumorigenesis and progression through transcriptional up‐regulation of *Nanog*.

## 1 INTRODUCTION

High mortality rate of lung cancer remains unchanged over the past several decades despite the advances in lung cancer treatment, in particular, the targeted therapies.^1^ Non‐small‐cell lung cancer (NSCLC) represents approximately 85% of all new lung cancer diagnoses, including adenocarcinoma, large‐cell carcinoma, and squamous cell (epidermoid) carcinoma.^2^ The low 5‐year survival rate of lung cancer (less than 17%) was attributed to late‐presentation, the lack of druggable targets, development of drug resistance to therapy, and high incidence of systemic metastasis.^3^, ^4^ Therefore, there is an urgent need to identify new diagnostic and/or therapeutic targets for lung cancer management. Accumulating evidence suggests that lung cancer progression might be driven by cancer stem cells (CSCs) that are more metastatic and refractory to conventional chemotherapeutics.^5^

CSCs are a subset of cancer cells that possess the self‐renewal capacity of normal stem cells (NSCs) and differentiate into heterogeneous lineages of cancer cells that comprise the tumor.^6^ The existence of leukemia CSCs was first identified (CD34+/CD38−) by Dick et al in 1994 which exhibit self‐renewal capacity, promotion of acute myeloid leukemia progression, chemoresistance and recurrence.^7^, ^8^, ^9^ Subsequent studies have identified CSCs in solid tumors including breast cancer,^10^ brain tumor,^11^ head and neck squamous cell carcinoma,^12^ pancreatic cancer,^13^ and lung cancer.^14^ CSCs isolated from clinical specimens were mostly obtained by flow cytometry sorting using cell‐surface markers. The drawbacks of this method of CSCs purification are the low yield of CSCs prohibiting in depth mechanistic investigation, and the heterogeneity among different clinical samples. To overcome this technical obstacle, we have developed an alternative approach for CSCs isolation and purification,^15^ and derive a distinct and stable sub‐population of cells within the Lewis lung cancer cells (LLCs) that employed large symmetric division for self‐renewal (LLC‐SD). We used LLC‐SD for mechanistic investigation in our present study.

The orphan nuclear receptor *Nr5a2*, also known as LRH‐1, plays a vital role in normal differentiation and development, cholesterol transport, bile‐acid homeostasis, and steroidogenesis.^16^, ^17^ In addition, *NR5A2 *is involved in the maintenance of pluripotency in embryonic stem cells (ESCs) ,^18^ reprogramming of somatic cells into induced pluripotent stem cells (iPSCs)^19^, ^20^ and controlling neural stem cell fate decisions.^21^ In recent years, accumulating evidence has also shown the participation of *Nr5a2* in the pathogenesis of various tumors including breast,^22^, ^23^ pancreatic,^24^ colon,^25^ gastric,^26^ and hepatocellular^27^ cancers. However, its role in regulating CSC functions remains elusive. Only one recent report showed the association of *Nr5a2* with CSCs in pancreatic cancer^28^ without mechanistic investigation. One mechanism underlying *Nr5a2* regulation of the stemness in ESCs is achieved through its regulation of Nanog,^19^ a key regulator of the self‐renewal of ESCs.^29^

In this study, we have provided convincing evidence in vitro and in vivo demonstrating that *Nr5a2* can induce lung CSC properties and promote tumorigenesis and progression through transcriptional up‐regulation of *Nanog*.

## 2 MATERIALS AND METHODS

### 2.1 Bioinformatics analysis

The copy number gain of *Nr5a2* in lung adenocarcinoma and normal tissues was evaluated by publicly available Oncomine database (https://www.oncomine.org). The thresholds were set as following: *P* = 0.05; fold‐change, all; gene rank = 10%; and data type, DNA. The correlation between *Nr5a2* mRNA expression levels and prognosis of lung adenocarcinoma patients was assessed by Kaplan–Meier plotter database (http://kmplot.com/analysis/). Kaplan–Meier survival plot was computed for the overall survival (OS) and progression‐free survival (PFS), with the hazard ratio(HR) with 95% confidence intervals(CI) and logrank *p* value.

### 2.2 Cell lines and culture

Mouse Lewis lung carcinoma parental cell line (LLC‐Parental) was a gift from Dr Robert Hoffman (University of California San Diego). LLC‐Parental was cultured in dulbecco's modified eagle medium (DMEM) high glucose supplemented (Hyclone, USA) with 10% fetal bovine serum (FBS) (ExCell Bio, USA). The symmetrical division cell line generated from LLC‐Parental cell line (LLC‐SD) was maintained in DMEM/F12‐based normal stem cell media (Hyclone, USA), supplemented with 2% B27 (Gibco, USA). Both cell lines were cultured in humidified atmosphere containing 5% CO_2 _at 37°C.

### 2.3 RNA extraction and RT‐qPCR analysis

RNA was extracted by TRIZOL (Takara, Japan) according to the manufacturer's protocol. RT‐PCR was conducted using PrimeScript RT Master Mix (Takara, Japan) according to the manufacturer's instructions. The sequences of PCR primers are listed in Table 2.

**Table 1:** Characterization of orthotopic LLC‐SD tumorigenesis and metastatic progression

10^4^ cells (n = 7)	In situ metastasis				
	Lung (left)	Mediastinal lymph	Lung (right)	Thoracic cavity (left)	Thoracic cavity (right)
sh‐NC	5/7	2/7	0/7	2/7	1/7
sh‐Nr5a2	1/7	0/7	0/7	0/7	0/7

**Table 2:** Primers for RT‐qPCR

Gene name	Forward primers	Reverse primers
mouse *Nr5a2*	AAACGGGCAGTAACCCTCTT	CCACATTTCAGCAACAGCAG
mouse *Nanog*	TTAGAAGCGTGGGTCTTGGT	CCCTCA AACTCCTGGTCCTT
mouse *Aldh1a1*	ATACTTGTCGGATTTAGGAGG CT	GGGCCTATCTTCCAAATGAAC A
mouse *Klf4*	GGACCACCTTGCCTTACACA	GACTTGCTGGGAACTTGACC
mouse *Bmi1*	ATCCCCACTTAATGTGTGTCCT	CTTGCTGGTCTCCAAGTA ACG
mouse *Sox2*	AGGGCTGGGAGAAAGAAGAG	ATCTGGCGGAGAATAGTTGG
mouse *CK‐18*	CTGGAAACTGAGAACAGGAGAC	CTCAGGTCTTCGATGATCTTGA
mouse *Nestin*	TGTTCTTGTAACTGCCCTAGAG	GCATCTAAATGGTCAATCGCTT
mouse *Tbp*	AGGGATTCAGGAAGACCACA	ATGCTGCCACCTGTAACTGA
human *Nr5a2*	TGCGTGGAGGAAGGAATAAG	TTGGATCACCTGAGACATGG
human *Nanog*	ACACTGGCTGAATCCTTCCTCTCC	CGCTGATTAGGCTCCAACCATACTC
human *Tbp*	TATAATCCCAAGCGGTTTGC	CACAGCTCCCCACCATATTC

### 2.4 siRNA transient interference assay

Two different siRNA duplexes targeting *Nr5a2* and negative control siRNA (siNC) were purchased from GenePharma (GenePharma, Co., Ltd, Shanghai, China). The sequences of the siRNAs are as follows: 5′‐GCUCACCUGAGUCAAUGAUTT‐3′ (si*Nr5a2*‐1), 5′‐CCUCUGCAAUUCAGAACAUTT‐3′ (si*Nr5a2*‐2), 5′‐UUCUCCGAACGUGUCACGUTT‐3′ (siN.C.). Small interfering RNA (siRNA) assay was performed and optimized using Lipofectamine2000 (Invitrogen, USA) and MEM medium (Gibco, USA) according to the manufacturer's protocol. For each siRNA transfection, 5 μL siRNA and 5 μL Lipofectamine2000 was diluted in 200 μL MEM medium respectively, and incubated for 5 minutes. Thereafter, the commixture was incubated for 30 minutes at room temperature, added to each cell well and the 6‐well plates were returned to the incubator and let alone for 48 hours.

### 2.5 Western blotting

Cells were lysed with radio immunoprecipitation assay buffer (Beyotime, China). Total protein concentration was determined by bicinchoninic acid method and the final concentration was adjusted to 5 μg/μL. Twenty‐five‐micrograms of protein was first separated on 10% SDS‐polyacrylamide (Beyotime, China) gels and electro‐transferred to a PVDF membrane (Bio‐Rad). The membrane was blocked in 5% BSA (Bio‐Rad, USA) and incubated with the primary antibody at 4°C overnight. Then, the membrane was incubated with the horseradish peroxidase‐conjugated secondary antibody. The resultant protein bands were visualized using the gel electrophoresis imager (Bio‐Rad). The primary antibodies were used at the following working dilutions: anti‐NR5A2 (1:1000 dilution, Santa Cruz, USA), and anti‐GAPDH (1:5000 dilution, Proteintech, USA).

### 2.6 Flow cytometry

The LLC‐SD‐siN.C. and LLC‐SD‐si*Nr5a2* (LLC‐SD‐si*Nr5a2*‐1/LLC‐SD‐si*Nr5a2*‐2) cells were analyzed for CD133 cell‐surface marker expression by flow cytometry according to the manufacturer's protocol. The primary antibody was used at the following working dilutions: anti‐CD133 antibody (0.2 μg 18470‐1‐AP, Proteintech, USA), and the secondary antibody: CL‐488‐conjugated Affinipure Goat Anti‐Rabbit lgG (H + L) (1:100‐1:500 dilution, Proteintech, USA).

### 2.7 Serial spheroid formation assay

Serial spheroid formation assay was done in the 6‐well plate format (Thermo, USA). A total of 1000 cells were suspended in 2 mL of medium and seeded to each well. After 5 days of incubation, the primary spheroids were dissociated, counted under the microscope and diluted to a density of 500 cells/mL by limiting dilution assay. A total of three rounds of this assay were performed.

### 2.8 Soft agar colony formation assay

A total of 200 cells were suspended in 1 mL of medium containing 0.2% agar (Sigma, USA) (to prevent cell aggregation) and were planted in 6‐well plates (Thermo, USA). One‐milliliter fresh media was added on the top of the agar layer. After 7 days of incubation, clonogenic spheroids that consisting of a minimum of 50 cells were counted under microscopy. 0.05% crystal violet was used to stain the colony forming units, and photographs of the 6‐well plates were taken.

### 2.9 Single‐cell cloning assay

The single‐cell suspensions were prepared at the concentration of 10 cells/mL by limiting dilution. Hundred‐microliter diluted suspensions was added to every well in 96‐well plates (Thermo, USA). Single‐cell plating was confirmed under microscopy and wells containing only one cell were marked. After 10 days of incubation, colonies that consisting of a minimum of 50 cells were counted under microscopy. Single‐cell cloning efficiency was calculated.

### 2.10 Lentiviral short‐hairpin RNA (shRNA) construction and transfection

The lentivirus plasmid PLL3.7 (Addgene, USA) was constructed with shRNA specific for *Nr5a2 *or negative control (GenePharma, China) after enzyme digestion. Recombinant lentiviruses expressing *Nr5a2* shRNA or negative control shRNA were obtained by plasmid transformation. Lentivirus was packaged in 293T cell line using the VSVG, pMDLg/pRRE and RSV‐REV (Addgene, USA), as well as Lipofectamine 2000 (Invitrogen, USA). Medium containing lentivirus was collected and filtered through 0.22 μM filter (Millipore, USA) after 48 hours. Fresh filtered virus containing medium was used for LLC‐SD cell transfection or stored at −80 °C for future use. LLC‐SD cells were infected with lentivirus and polybrene (Sigma, USA) added with the final concentration of 8 μg/mL.

### 2.11 Animals

Six to eight weeks old female BALB/c nude mice or C57BL/6 were provided by the Chongqing national biological industry base experimental animal center of Chongqing Medical University. All animal experiments were performed in accordance with the animal welfare and institutional ethical guidelines of Chongqing Medical University and with the protocol approved by the Institutional Review Board of Chongqing Medical University.

### 2.12 Subcutaneous tumor transplantation assay in BALB/c nude mice

Single‐cell suspensions were mixed with equal volume of Growth Factor Reduced Matrigel Matrix (Corning, USA). Hundred‐microliters mixture containing 1 × 10^4^ cells was injected subcutaneously into the hind leg of BALB/c nude mice. Tumor growth was monitored and tumor volume was measured every 2 days. Mice were sacrificed and photographed when tumor volume reached 1 cm^3^. Tumor volume was calculated as V = (length × width^2^)/2.

### 2.13 Orthotopic tumor transplantation of C57BL/6 mice

The single‐cell suspensions were mixed with equal volume of Growth Factor Reduced Matrigel Matrix (Corning). 0.20 μL mixture containing 1 × 10^4^ cells was injected orthotopically into the left lobe of the lungs of C57BL/6 mice as described previously.^15^ For tumorigenesis and progression experiments, mice were dissected on day 14 to determine the growth of the orthotopic tumors at the site of injection and the extent of thoracic metastasis. For the survival experiments, the death time of every mouse was recorded after orthotopic tumor transplantation until the last mouse of the group was dead.

### 2.14 Chromatin immunoprecipitation (ChIP) assay

ChIP assay was performed to detect the molecular interactions of *Nr5a2* with the promoter of *Nanog* according to manufacturer's instruction (Beyotime, China). Briefly, LLC‐SD cells were cross‐linked with 1% formaldehyde for 10 minutes at 37°C and cross‐linking was stopped by adding glycine solution for 5 minutes at room temperature. Subsequently, the lysed cells were isolated and sonicated on ice to shear DNA into fragments of 200 bp to 1 kb. Chromatin complexes were immunoprecipitated by incubating with anti‐NR5A2 or control anti‐IgG antibodies at 4°C overnight with rotation. The input DNA was isolated from the sonicated lysates before immunoprecipitation as a positive control. After washing and de‐crosslinking, purified DNA was detected by qPCR with primers listed in Table 3.

**Table 3:** Primers for ChIP‐qPCR

Region	Forward primers	Reverse primers
29‐43	AGACAGAAGCAGGTGGGTCTC	GACAGAGTTTCTCTATGTAGCCCCGG
999‐1013	TGAAACAAGAAATGGCTGCTTT	GGCTGGCCTTGAACTCAGAA
1028‐1042	TGTCTAATTGAAACAAGAAATGGCTGC	TGGTTGGTTGGTTGGTTTTTCGA
1844‐1858	CAGTGGTGGCACATACAGGC	GGGGTTGGTGGTGTTTGTTTGA
2143‐2157	GGCCCTTCCCTCTCTGCTTA	CCACATCCCAAGTTCAAAGTTTGC
Negative control	TCAAAGTTGTCAGAGGAGGGC	TGAACCCTGGTCCTCTGGAA

### 2.15 Transient overexpression assay

mNanog pcDNA3.1‐3xFlag‐C and negative control were purchased from Youbio (Youbio, Co., Ltd, Hunan, China). Transient overexpression assay was performed and optimized using Lipofectamine2000 (Invitrogen, USA) and MEM medium (Gibco, USA) according to the manufacturer's protocol.

### 2.16 Clinical samples

Paraffin‐embedded lung adenocarcinoma tissues were obtained from the Pathology Department of the Affiliated Hospital of Southwest Medical University. Patient consent forms were obtained according to protocols approved by the Institutional Review Board of the Affiliated Hospital of Southwest Medical University. Samples were divided according to the UICC/AJCC tumor, lymph node and metastasis (TNM) classification of lung cancer (version 8.0) into‐IIB stages as early stage, and III‐IVstages defined as advanced stage.

### 2.17 Statistical analysis

Statistical analysis was performed by Graphpad Prism version 7.0. All values were expressed as mean ± SEM (n ≥ 3). ANOVA and Student's independent *t* test were performed to obtain *P*‐values. *P < *0.05 is considered statistically significant. *P < 0.05, **P < 0.01, ***P < 0.001. The Kaplan–Meier survival curves were used for OS analysis. Correlation of *Nr5a2* and *Nanog* expression was calculated with Spearman correlation coefficient formula by SPSS 13.0.

## 3 RESULTS

### 3.1 The clinicopathologic and prognostic importance of *Nr5a2* expression in human lung adenocarcinoma

**Figure 1:**
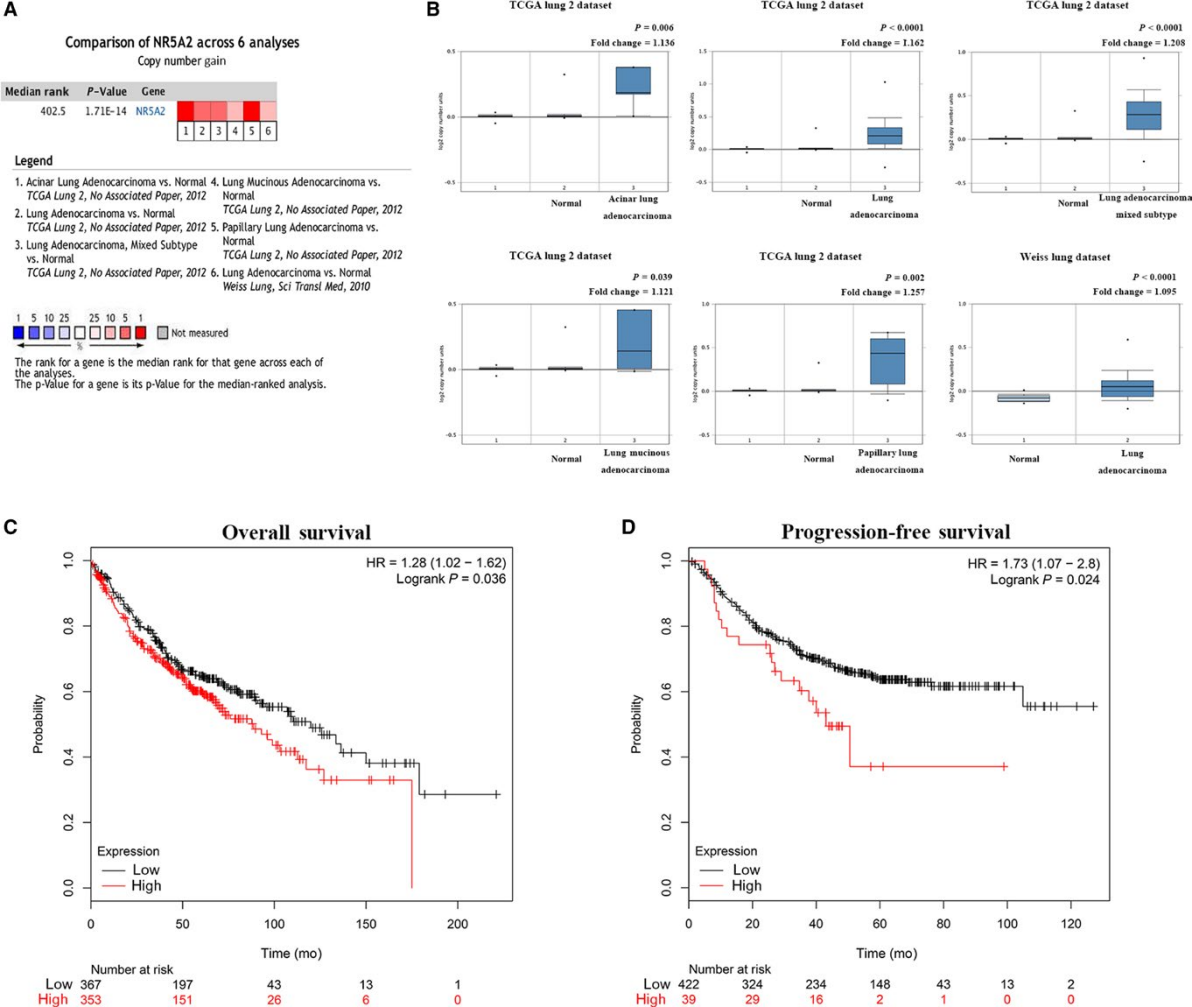
The bioinformatic data mining analysis of the prognostic significance of *Nr5a2 *expression in human lung adenocarcinoma. (A) Meta‐analysis of six Oncomine datasets on *Nr5a2* copy number gain in lung cancer versus normal tissues. (B) Comparison of the copy number of *Nr5a2 *in normal (left plot) and cancer tissues (right plot). The analysis was conducted in acinar lung adenocarcinoma, lung adenocarcinoma mixed subtype, lung adenocarcinoma, lung mucinous adenocarcinoma, and papillary lung adenocarcinoma (*P* < 0.05). (C) Prognostic value of *Nr5a2 *on the overall survival from the Kaplan–Meier plotter database (*P* < 0.05). (D) Prognostic value of *Nr5a2 *on progression‐free survival from the Kaplan–Meier plotter database (*P < *0.05). HR, hazard ratio

To determine the clinical relevance of *Nr5a2* in human lung cancer, we analyzed the copy number of *Nr5a2* between lung adenocarcinoma and normal lung tissues using Oncomine database. A total of six studies, derived from TCGA lung2 datasets and GEO (GSE25016)^30^ datasets, were used for the bioinformatics analysis (Figure 1A, *P < *0.001). *Nr5a2* was significantly overexpressed due to copy number increase in lung adenocarcinoma (n = 427) compared to normal lung tissues (Figure 1B, n = 449, *P < *0.05). Kaplan–Meier survival analysis revealed that *Nr5a2* confers poor prognosis in human NSCLC for both OS as well as the PFS (*P < *0.05, Figure 1C,D).

### 3.2 *Nr5a2* expression is elevated in LLC‐SD CSC cells

**Figure 2:**
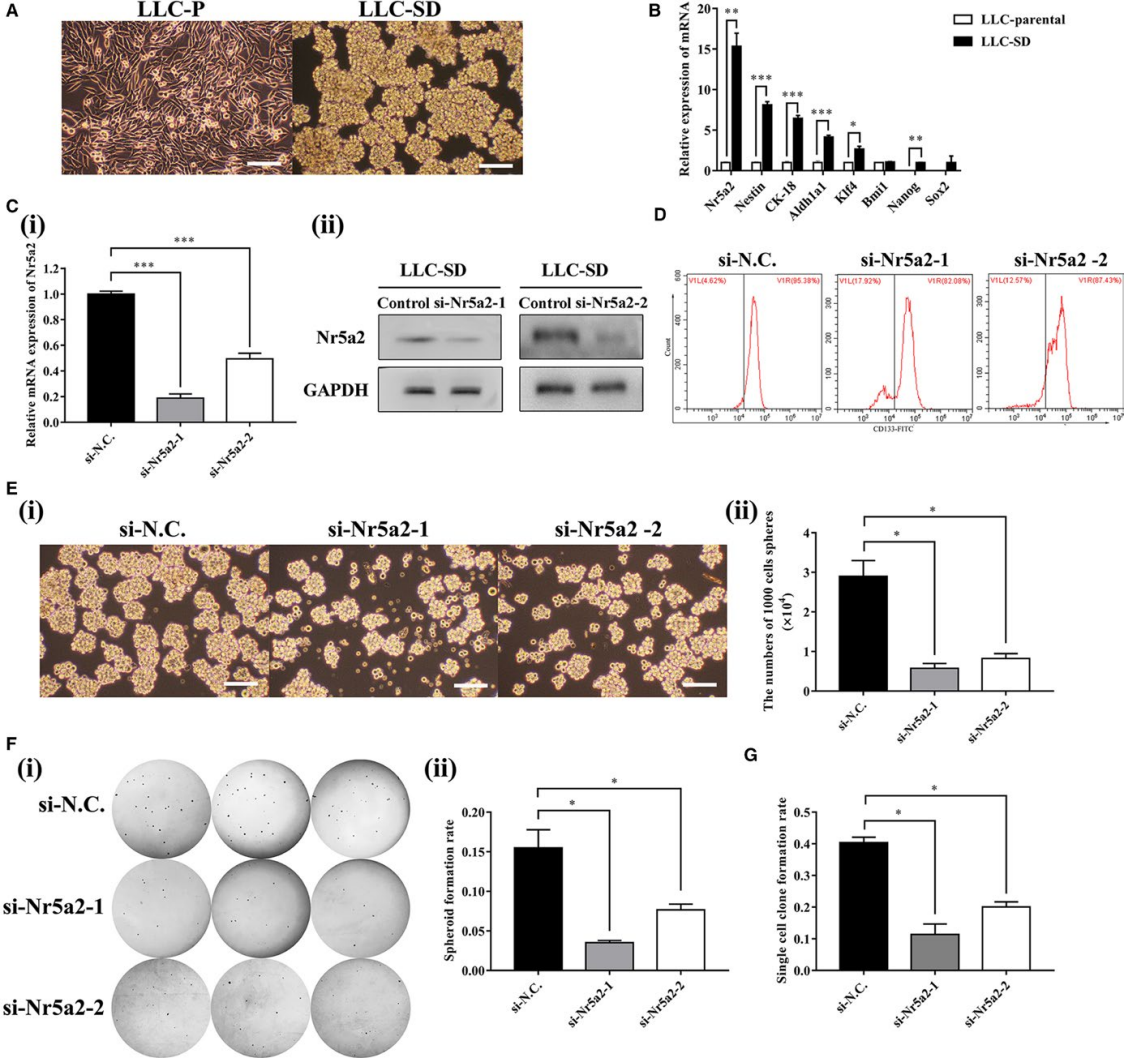
*Nr5a2*promotes LLC‐SD self‐renewal in vitro. (A) Cellular morphology of mouse Lewis lung carcinoma parental cells (LLC‐P) compared to mouse Lewis lung carcinoma symmetric division cells (LLC‐SD). Scale bars, 120 μm. (B) RT‐PCR analysis of mRNA expression of stemness markers (*Nr5a2, Nestin*, *CK‐18*, *Aldh1a1*, *Klf4*, *Bmi1*, *Nanog*, and* Sox2*). *TBP* was used as the endogenous control, *P < 0.05, **P < 0.01 and ***P < 0.001. (C)(i) Efficiency of two *Nr5a2* siRNAs (si*Nr5a2*‐1 and si*Nr5a2*‐2) interference determined by RT‐qPCR, *TBP* was used as the endogenous control, ***P < 0.001 and by Western blot analysis, using GAPDH as an internal control (ii). (D)the expression of CSCs marker CD133 in LLC‐SD‐siN.C. and LLC‐SD‐siNr5a2(siNr5a2‐1 and siNr5a2‐2) used by flow cytometry (E) (i) The morphology of spheroid formation in LLC‐SD cells transfected with negative control siRNA (siN.C.) and with specific siRNAs of *Nr5a2* (si*Nr5a2*‐1 and si*Nr5a2*‐2). Scale bars, 120 μm. (ii) Quantitative analysis of the number of spheroids in 6‐well colony formation assay. Data are presented as mean ± SEM of three independent experiments, *P < 0.05. (F) (i) The morphology of soft agar spheroid formation using LLC‐SD‐siN.C., LLC‐SD‐si*Nr5a2*‐1 and LLC‐SD‐si*Nr5a2*‐2 cells. (ii) Analysis of spheroid formation rate. Data are presented as mean ± SEM of three independent experiments, *P < 0.05. (G) Quantitative analysis of single‐cell cloning formation from LLC‐SD cells transfected with negative control siRNA (siN.C.) and knockdown of *Nr5a2* expression with two siRNAs (si*Nr5a2*‐1 and si*Nr5a2*‐2) in which 96 wells were assessed respectively. Data are presented as mean ± SEM of three independent experiments, *P < 0.05

Eight rounds of selection for stable spheroid‐forming floating cells followed by five successive rounds of single‐cell cloning assay resulted in the isolation and purification of a subcomponent of spheroid‐forming LLC cells from the parental mouse Lewis lung carcinoma cell (LLC‐Parental) culture, which exhibited the round and undifferentiated morphology of NSCs and underwent symmetrical division (LLC‐SD) (Figure 2A).

Using RT‐qPCR, we found the expression of embryonic stem cell markers (*Nr5a2*, *Nestin*, *CK‐18*, *Aldh1a1*, *Klf4*, *Nanog*) was significantly increased in the LLC‐SD cells compared to that in the LLC‐Parental cells (Figure 2B, 15.33 ± 1.63 fold, *P < *0.01). Among all markers assessed, the elevation of *Nr5a2* mRNA expression was most pronounced (Figure 2B).

### 3.3 siRNAs interference of *Nr5a2* expression suppresses spheroid formation and cloning efficiency in vitro

To investigate the relationship between elevated* Nr5a2* and changes in the stem cell properties of LLC‐SD cells, we first inhibited *Nr5a2* expression by transient siRNA‐1 and siRNA‐2 interference and verified its down‐regulation at both transcriptional (81.20 ± 0.03% and 50.6 ± 0.04%, respectively, Figure 2C‐i, *P < *0.001) and translational levels (Figure 2C**‐**ii). Meanwhile, the flow cytometry was used for the analysis of expression of CSCs marker CD133 in LLC‐SD‐siN.C. and LLC‐SD‐si*Nr5a2* (si*Nr5a2*‐1/si*Nr5a2*‐2) cells. The results revealed that the LLC‐SD‐si*Nr5a2*‐1 and LLC‐SD‐si*Nr5a2*‐2 cells have lower level of CD133 (82.08% and 87.43%, respectively) compared to the LLC‐SD‐NC cells (95.38%) (Figure 2D**)**.

We then examined spheroid formation efficiency by the serial spheroid formation assay in 6‐well plate after siRNA interference. Thousand cells were serially passaged to form second round (also referred as P2) and third round (P3) spheroids (Figure S1). Both of the si*Nr5a2* (si*Nr5a2*‐1/si*Nr5a2*‐2) interference altered the size of the spheroids and cell morphology, and greatly reduced the number of spheroids (Figure 2E**‐**i and Figure 2E**‐**ii). However, such inhibitory effect was observed only in the first round of the assay, likely due to transient inhibition of *Nr5a2*.

To further verify whether *Nr5a2 *could promote stemness properties, soft agar colony formation assay and single‐cell cloning formation assay which are the two assays widely used in stem cell research for the assessment of self‐renewal, were performed. The number of clonogenic spheroids scored on day 7 was significantly lower in LLC‐SD‐si*Nr5a2*‐1 (0.04 ± 0.00) and LLC‐SD‐si*Nr5a2*‐2 (0.08 ± 0.00) cells than that in the negative control (0.16 ± 0.02) (Figure 2F**‐**i and Figure 2F‐ii, *P < *0.05, n = 3). Similarly, single‐cell cloning rate of LLC‐SD‐si*Nr5a2*‐1 (0.11 ± 0.03) and LLC‐SD‐si*Nr5a2*‐2 (0.20 ± 0.02) cells was also significantly lower than that of the control cells (0.40 ± 0.02, *P < *0.05, n = 3, Figure 2G). These observations indicated that *Nr5a2* promoted LLC‐SD self‐renewal ability and clonogenic activity in vitro.

### 3.4 *Nr5a2 *interference inhibits tumorigenicity in vivo

**Figure 3:**
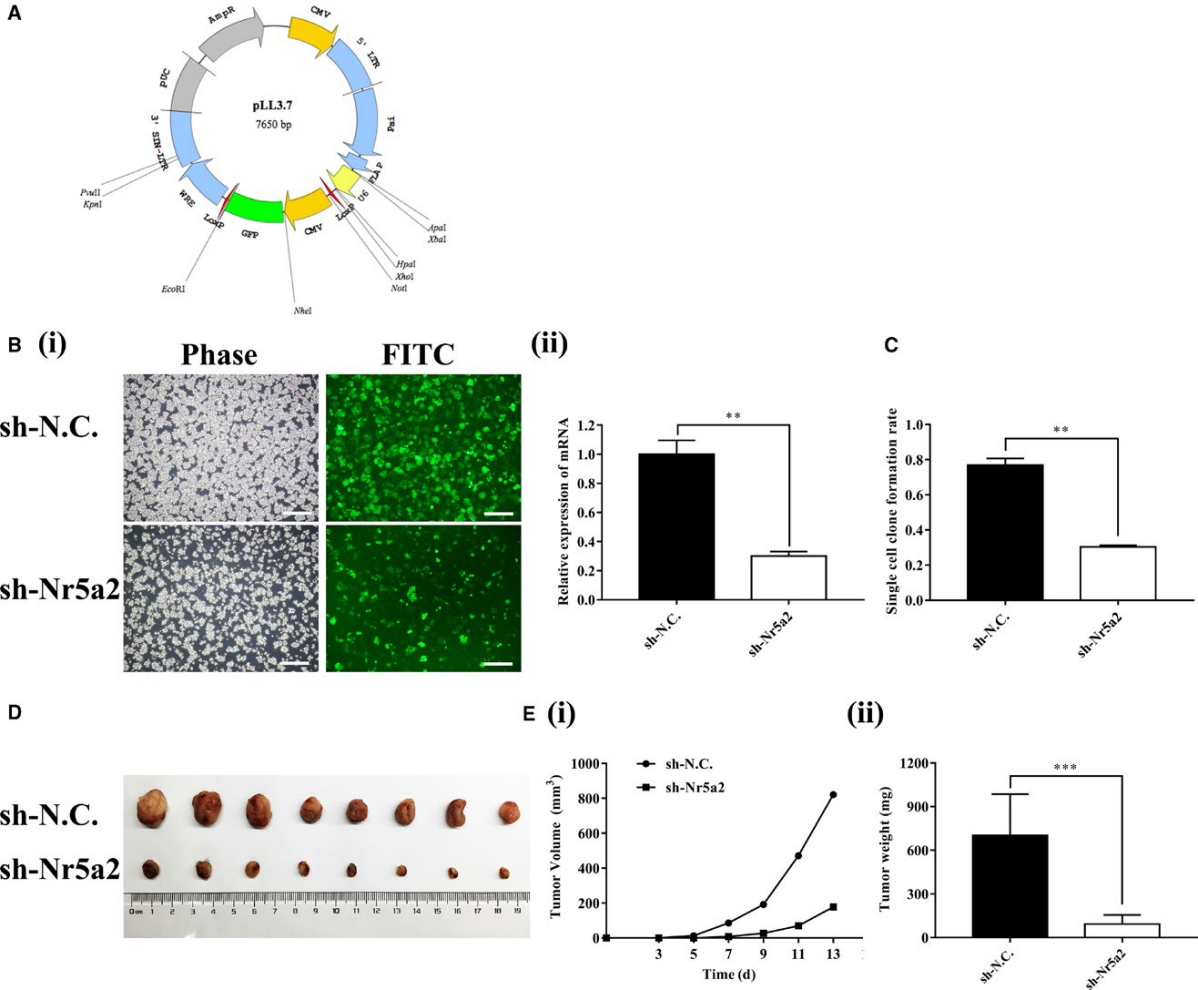
*Nr5a2* promotes lung adenocarcinoma tumorigenesis in nude mice. (A) pLL3.7 Vector plasmid profile map. (B)(i) Morphology of LLC‐SD cells infected by pLL3.7 lentivirus with sh*Nr5a2* and negative control shRNA. Scale bars, 120 μm. (ii) Interference efficiency of *Nr5a2* shRNA determined by RT‐qPCR, *TBP* was used as the endogenous control, **P < 0.01. (C) Analysis of single‐cell cloning formation from LLC‐SD cells infected with LLC‐SD‐sh*Nr5a2* and the control cells. Data are presented as mean ± SEM of three independent experiments, **P < 0.01. (D) Tumor formation in nude mice following injection of LLC‐SD‐sh*Nr5a2 *or the control cells. (E) Tumor growth curves of LLC‐SD‐sh*Nr5a2* and control cells in nude mice. (i) Tumor volume. (ii) Tumor weight, ***P < 0.001

Given the essential role of *Nr5a2 *in regulating LLC‐SD self‐renewal activity in vitro, we conducted xenograft transplantation assay in nude mice to investigate the importance of *Nr5a2* in lung cancer tumorigenesis. To ensure sustained *Nr5a2* inhibition in vivo, *Nr5a2 *expression was stably inhibited by lentiviral vector‐mediated gene knockdown (Materials and methods) in LLC‐SD cells (Figure 3A and Figure 3B**‐**i). An effective inhibition of *Nr5a2* expression in LLC‐SD‐sh*Nr5a2* cells was confirmed by RT‐qPCR analysis (70.00 ± 0.03%, *P < *0.01, n = 3; Figure 3B**‐**ii). The inhibition of single‐cell cloning rate upon *Nr5a2* stable inhibition confirmed the role of *Nr5a2 *in promoting LLC‐SD self‐renewal (Figure 3C, *P < *0.01).

10^4^ LLC‐SD‐sh*Nr5a2 *or control cells were injected subcutaneously into each side of the hinder leg of nude mice. Measurable tumors were formed after 2 weeks of tumor cell inoculation (8/8 sites). However, LLC‐SD‐sh*Nr5a2* tumors were significantly smaller than that of LLC‐SD‐NC tumors (Figure 3D). The growth and weight of LLC‐SD‐sh*Nr5a2* tumors were inhibited compared to LLC‐SD‐shN.C. control tumors (Figure 3E**‐**i and Figure 3E**‐**ii, *P < *0.001). Taken together, *Nr5a2* promotes lung CSCs tumorigenesis.

### 3.5 *Nr5a2 *interference inhibits orthotopic LLC‐SD tumorigenesis and progression

**Figure 4:**
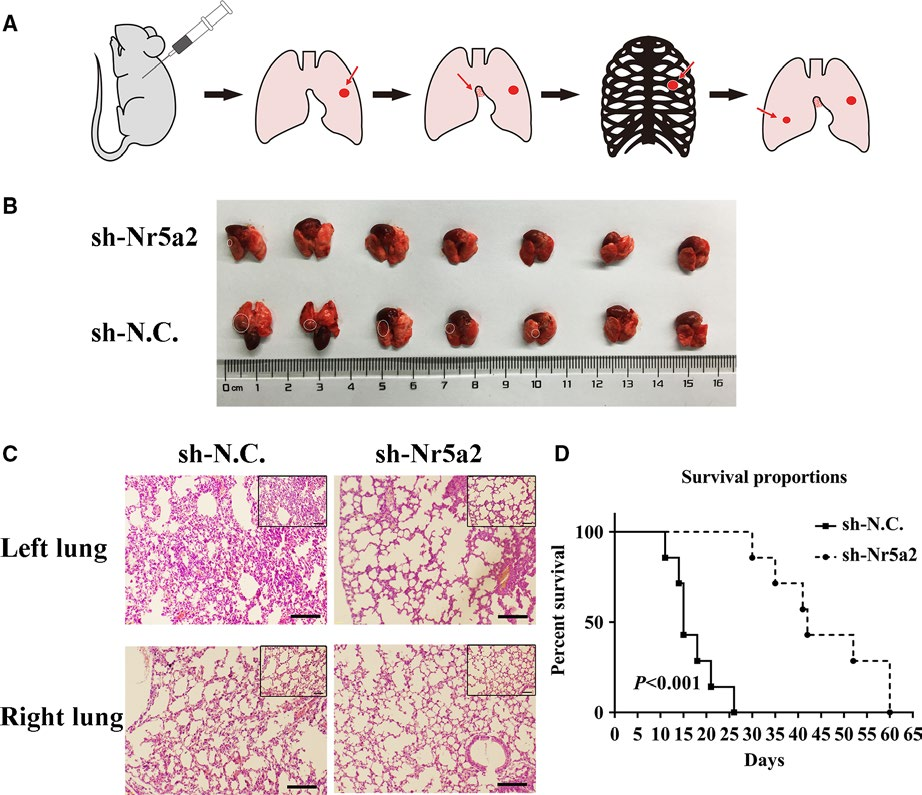
The effect of *Nr5a2* inhibition on tumorigenicity and progression in C57BL/6 mice with left lung orthotopic implantation. (A) A schematic overview of orthotopic implantation of LLC‐SD cell lines in C57BL/6 mice. (B) Images of left lung orthotopic nodules in C57BL/6 mice injected with LLC‐SD‐sh*Nr5a2 *or control cells (n = 7). (C) Immunohistochemistry analysis of orthotopic tumors derived from LLC‐SD‐sh*Nr5a2* and control cells transplanted to the lung of C57BL/6 mice. Scale bars = 120 μm, the black box indicates the enlarged area, bar = 60 μm. (D) In vivo survival assay (Materials and methods)

Prior to this study, we have developed a clinically relevant syngeneic orthotopic mouse model of lung cancer which allowed the evaluation of tumorigenicity and metastasis of LLC‐SD with *Nr5a2* depletion in C57BL/6 mice (Figure 4A).^15^ We employed this model to evaluate the relationship between the self‐renewal promoting effect of *Nr5a2* that we observed in vitro (Figures 2E and 3C) and the cancer biological properties in vivo.

10^4^ LLC‐SD‐shN.C. control cells or LLC‐SD‐sh*Nr5a2* cells were injected in the left lung of C57BL/6 mice, respectively (Materials and methods, n = 7).^15^ All mice were sacrificed on day 14 post tumor cell injection. Orthotopic tumor growth and metastatic progression in the thoracic cavity were examined and recorded. At the site of tumor cell injection, tumors were developed in five of seven mice injected with LLC‐SD‐shN.C. cells and in one of seven mice injected with LLC‐SD‐sh*Nr5a2* cells. In mice injected with LLC‐SD‐shN.C. cells, visible metastatic foci were found at left thoracic cavity (2/7 mice), right thoracic cavity (1/7 mice), and mediastinal lymph nodes (2/7 mice). In contrast, no thoracic metastases were observed in mice injected with LLC‐SD‐sh*Nr5a2* cells (Table 1 and Figure S2).

LLC‐SD‐shN.C. and LLC‐SD‐sh*Nr5a2* tumors on the left lung were removed and analyzed by hematoxylin and eosin (HE) staining. HE staining of left lung tissue from LLC‐SD‐shN.C. tumor showed destroyed alveoli and poorly differentiated hyperchromatic tumor cells with prominent nucleoli. Vascular invasion of tumor cells was also evident (Figure 4C). Meanwhile, HE staining of right lung tissue from LLC‐SD‐shN.C. tumor showed normal lung structure. Only few scatted tumor cells were found. However, no typical lung carcinoma‐like morphology was observed in the left lung tissue and no tumor cells were found in the right lung from LLC‐SD‐sh*Nr5a2* group (Figure 4B‐C).

We also conducted mouse survival assay upon orthotopic injection of 10^4^ LLC‐SD‐sh*Nr5a2 *and LLC‐SD‐shN.C. cells (Materials and methods, n = 7). Death of the animal was recorded and the assay was terminated on day 60 post tumor cell transplantation. Mice in the LLC‐SD‐shN.C. group died from 11th day until the 26th day. In contrast, the mice in the LLC‐SD‐sh*Nr5a2* group died from 30th day until the 60th day (Figure 4D). The survival curve of mice injected with LLC‐SD‐sh*Nr5a2* cells was significantly right‐shifted (Figure 4D).

Based on the above observations, we conclude that the self‐renewal promoting function of *Nr5a2* observed in vitro is essential for LLC‐SD tumorigenesis and progression in vivo.

### 3.6 *Nr5a2* regulates *Nanog* expression through directly binding to its promoter

**Figure 5:**
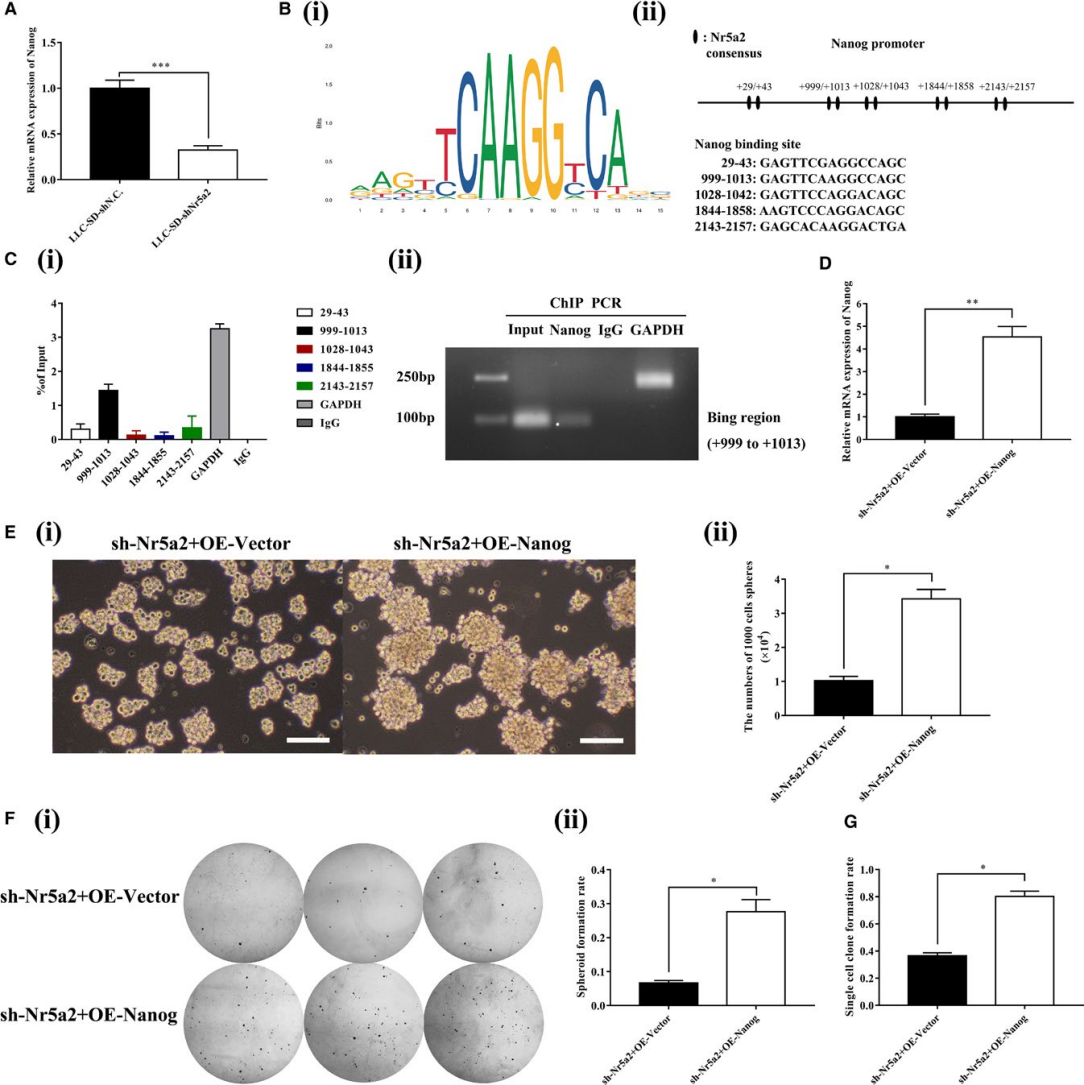
*Nr5a2 *directly targets *Nanog* transcription to promote stemness of LLC‐SD in vitro. (A) Measurement of the downstream stem cell‐related genes of *Nr5a2 *by RT‐qPCR, *TBP* was used as the endogenous control, ***P < 0.001. (B) (i) Motif of *Nr5a2* binding sites. (ii) Binding sites of *Nr5a2* in the promoter region of *Nanog*. (C)(i) ChIP analysis of the interaction of *Nr5a2* with *Nanog* promoter. Data are presented as mean ± SEM of three independent experiments. (ii) Agarose gel electrophoresis confirmed the binding site of *Nr5a2* to the *Nanog* promoter. GAPDH was used as a positive control. (D) Expression of *Nanog *in LLC‐SD‐sh*Nr5a2*+OE‐Vector and LLC‐SD‐sh*Nr5a2*+OE‐*Nanog *cells by RT‐qPCR, *TBP* was used as the endogenous control, **P < 0.01. E(i) The morphology of spheroid formation in LLC‐SD‐sh*Nr5a2*+OE‐Vector and LLC‐SD‐sh*Nr5a2*+OE‐*Nanog *cells. Scale bars, 120 μm. (ii) Quantitative analysis of the number of spheroids formation assay. Data are presented as mean ± SEM of three independent experiments, *P < 0.05. F(i) the morphology of soft agar spheroid formation using LLC‐SD‐sh*Nr5a2*+OE‐Vector and LLC‐SD‐sh*Nr5a2*+OE‐*Nanog *cells. (ii) Analysis of spheroid formation rate. Data are presented as mean ± SEM of three independent experiments, *P < 0.05. (G) Quantitative analysis of single‐cell cloning formation from LLC‐SD‐sh*Nr5a2*+OE‐Vector and LLC‐SD‐sh*Nr5a2*+OE‐*Nanog *cells in which 96 wells were assessed respectively. Data are presented as mean ± SEM of three independent experiments, *P < 0.05

Since *Nr5a2* is a transcriptional co‐activator, it may promote LLC‐SD CSC stem cell like properties and in vivo cancer biology characteristics by transcriptional activation of gene(s) that are regulators of NSCs and CSCs. We determined the expression of known stem cell‐related genes in the LLC‐SD cells after *Nr5a2 *stably inhibited by RT‐qPCR (data not shown). Down‐regulation of *Nanog* expression upon *Nr5a2 *inhibition was observed (Figure 5A, *P* < 0.01). We thus hypothesized that *Nanog*, the most significantly down‐regulated gene, could be a *bona fide *transcriptional target of *Nr5a2*.

To test this hypothesis, we used the motif of *Nr5a2 *and promotor sequence of *Nanog* to predict transcriptional factor binding site by bioinformatics in JASPAR database (Figure 5B**‐**i). We found five putative *Nr5a2* response elements which localized in the +29 ~ +43, +999 ~ +1013, +1028 ~ +1042, +1844 ~ +1858, +2143 ~ +2157 region of *Nanog* promoter, respectively (Figure 5B**‐**ii).

Next, we carried out chromatin immunoprecipitation (ChIP) assay to confirm the bioinformatics analysis. ChIP primers were designed to amplify promoter regions containing the predicted putative binding sites of *Nr5a2*, and the distal region primer was used as a negative control (Table 3). We incubated the nuclear extracts of LLC‐SD cells in the presence of anti‐*Nr5a2 *antibody, anti‐RNA Polymerase II antibody (as a positive control) or syngeneic IgG (as a negative control). Quantitative PCR analysis showed that *Nr5a2* localized in the +999 ~ +1013 region of *Nanog *promoter was responsible for the majority of its transcriptional activation activity (Figure 5C**‐**i). In addition, DNA gel electrophoresis was used to confirm the finding by ChIP analysis (Figure 5C**‐**ii). In summary, these results indicate that *Nr5a2 *directly activates *Nanog* transcription.

### 3.7 Overexpression of *Nanog *in LLC‐SD‐sh*Nr5a2 *cells restored stemness properties of LLC‐SD cells in vitro

To confirm that *Nanog* is indeed the downstream target that mediating the stemness maintenance property of *Nar5a2*, the expression of *Nanog* was restored in *Nr5a2* stably inhibited LLC‐SD‐sh*Nr5a2* cells by overexpressing of *Nanog *(Figure 5D). The restoration of *Nanog *expression reversed self‐renewal ability and clonogenic activity in LLC‐SD‐sh*Nr5a2* cells, measured by serial spheroid formation assay (Figure 5E), soft agar colony formation assay (Figure 5F) and single‐cell cloning formation assay (Figure 5G). These results indicate that *Nr5a2 *promotes lung CSCs stemness by directly targeting transcriptional activation of *Nanog*.

### 3.8 High expression of *Nr5a2 *in advanced NSCLC paraffin‐embedded tissues correlated with *Nanog *levels

**Figure 6:**
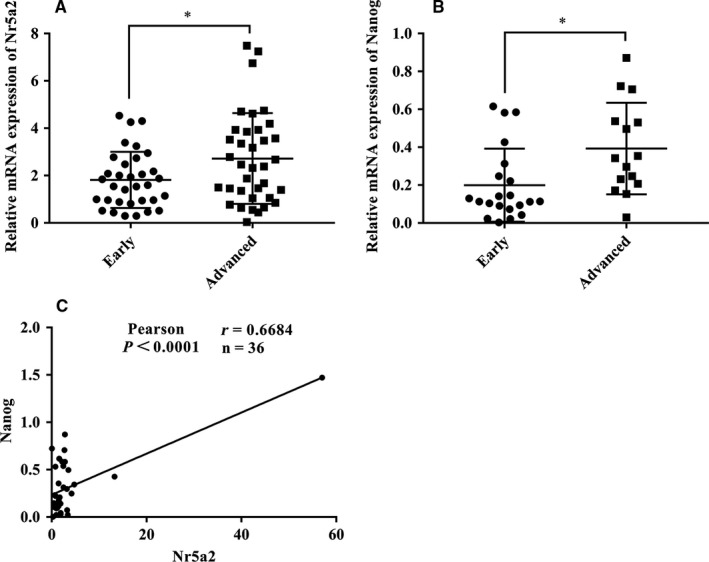
Expression of *Nr5a2 *in clinical samples and correlations with *Nanog* expression. (A) Scatter dot plot shows the relative levels of *Nr5a2 *in lung adenocarcinoma patients by RT‐qPCR *TBP* was used as an internal control, *P < 0.05. (B) Expression of *Nanog *was determined by RT‐qPCR in lung adenocarcinoma. *TBP *was used as an internal control, *P < 0.05. (C) Spearman test was performed to determine the correlation between *Nr5a2 *and *Nanog* in lung adenocarcinoma patients

To investigate whether *Nr5a2* and *Nanog* expressions have clinical implications in human cancers, we examined their expressions in NSCLC paraffin‐embedded tissues with distinct TNM stage by RT‐qPCR. A total of 69 NSCLC samples were collected for *Nr5a2* analysis, among which 33 cases were at early stage (stageⅠ~Ⅱ) and 36 cases were at advanced stage (stageⅢ~Ⅳ), respectively. Similarly, a total of 36 NSCLC samples were collected for *Nanog* analysis, among which 21 cases were at early stage and 15 cases were at advanced stage, respectively. Elevated levels of *Nr5a2 *and* Nanog* expression were both observed in advanced stage NSCLC samples compared to that in the early stage NSCLC samples (Figure 6A‐B). Linear regression analysis demonstrated a significant positive correlation between the mRNA expression of *Nanog* and *Nr5a2* (Figure 6C**,** *P* < 0.0001, r = 0.6684) in 36 cases of NSCLC patient samples. Collectively, these results are consistent with our findings in the LLC‐SD experimental model. More importantly, they suggest that in human NSCLC, *Nanog* might also be under the transcriptional regulation of *Nr5a2* to maintain the stemness properties and to promote NSCLC progression.

In summary, this study has provided new understanding of the function of *Nr5a2 *in regulating lung CSCs self‐renew which is reminiscent of its function in NSCs.

## 4 DISCUSSION

Prior to the present study, the role of *Nr5a2* in regulating CSCs function has not been established.^28^ Our knowledge of the promoting effect of *Nr5a2* on stem cell self‐renewal comes mostly from studies involving NSCs.^21^ A recent study reported that *Nr5a2* can substitute for *Oct4* and *Klf4* in ESCs and induced pluripotent stem cells (iPSCs) for the modulation of pluripotency and reprogramming.^31^ This study demonstrated for the first time that *Nr5a2 may *play a similarly important role in regulating the self‐renewal of lung CSCs derived from the syngeneic mouse Lewis lung adenocarcinoma LLC‐SD.

We made the following new findings that had not been previously reported:

First, the prognostic value of *Nr5a2* overexpression in human lung cancer has been evaluated and established. Kaplan–Meier plotter analysis was conducted using the Oncomine database. *Nr5a2 *amplification, observed in lung cancer tissues (Figure 1, 2, 3, 4, 5, 6A‐B), is associated with poor OS and PFS (Figure 1, 2, 3, 4, 5, 6C ‐D). This set of observations suggests that *Nr5a2* promotes human lung cancer progression. Future in‐depth mechanistic characterization will answer whether *Nr5a2* is a novel target for therapeutic development.

Second, generation of LLC‐SD lung CSC cellular model affords mechanistic investigation. The lack of stable cellular models of lung CSCs has hindered mechanistic research of lung CSCs. In a recent report,^15^ we presented a new method that differs from cell‐surface marker sorting for enriching and purifying CSCs which yielded the stable cellular model of lung CSC, that is, the LLC‐SD cell line that underwent symmetrical division (Figure 2A). We showed that *Nr5a2* was highly expressed in LLC‐SD cells compared to LLC‐P cells.

Third, using the LLC‐SD model, through transient and stable siRNA interference of *Nr5a2* expression, we provided convincing evidence for a regulatory role of *Nr5a2* in the maintenance of lung CSC self‐renewal and stem cell properties (Figure 2D**‐**G) in vitro. Further, using the syngeneic and orthotopic lung transplantation model we established and characterized,^15^ we evaluated the impact of alterations in stem cell properties upon *Nr5a2* interference observed in vitro on the cancer biology properties in vivo. The effects of *Nr5a2* in promoting LLC‐SD oncogenesis and metastatic progression, as well as the resultant shorter survival (Figures 3, 4 and Table 1) are consistent with but add new perspectives to the previously reported oncogenic activity of *Nr5a2* in NSCLC.^32^ This set of in vivo observations, made in the orthotopic and syngeneic model of lung cancer, have overcome the lack of proper animal models in CSC research for the evaluation of CSC cancer biology properties in vivo.

Fourth, *Nr5a2*’s regulatory role in promoting LLC‐SD self‐renewal is mediated by its direct transcriptional target *Nanog*. We showed that *Nr5a2 *could directly bind to the *Nanog *promoter to stimulate its expression (Figure 5A‐C). Furthermore, the direct targeting of *Nanog* by *Nr5a2* was confirmed by restoration of *Nanog* in *Nr5a2 *silenced LLC‐SD‐sh*Nr5a2* cells which successfully reversed the self‐renewal phenotype seen in LLC‐SD‐sh*Nr5a2*+OE‐*Nanog* cells in vitro (Figure 5E**‐**G). Consistent with our findings, a previous study had shown that *Nr5a2 *can replace *Oct4* in the reprogramming of murine somatic cells into pluripotent cells and enhance the reprogramming efficiency by directly regulates *Nanog*.^19^ However, in our studies, we did not observe significant changes in *Oct4* upon *Nr5a2* interference (data not shown). Few recent studies have shown the crucial role of *Nanog* in tumorigenesis of lung adenocarcinoma,^33^ gastric adenocarcinoma,^34^ and colorectal cancer.^35^ In our analyses, the elevated expression of *Nanog *was significantly correlated with the *Nr5a2* amplification (Figure 5A) and with elevated *Nr5a2* expression in NSCLC patient samples of advanced stage (Figure 6). These observations for the first time showed that *Nr5a2 *may be correlated with advanced TNM stages and poor prognosis by activating *Nanog *transcription. Hence, the diagnostic, prognostic, and therapeutic potential of *Nr5a2* in lung cancer merits further investigation.

The source of CSCs has been an important and unsolved key scientific issue in CSC research, in particular, whether somatic cells or NSCs could become the CSCs under malignant transformation. Firstly, recent reports showed that some of pluripotent genes could promote the self‐renewal of CSCs which maintain the stemness of ESCs and iPSCs such as *Oct4* and *Nanog*.^36^, ^37^ Our findings and others indicate that CSCs may utilize the self‐renewal pathways of the NSCs.^38^ Secondly, a study in the skin tumor model showed that only the skin stem cells, not the progenitor cells could give arise to skin CSCs to initiate tumor formation.^39^ This study argues that the source of CSCs, in rapidly regenerating skin, is likely the normal skin stem cells. And lastly, there is another school of thinking regarding the source of CSCs in human cancer as tumor cells that have the plasticity similar to somatic iPS.^40^ Since *Nanog* is a key regulator for the maintenance of stemness of NSCs as well for inducing the iPS state, our results can not differentiate the function of *Nr5a2* via *Nanog* on LLC‐SD whether through regulating the stemness of original stem cells enriched in LLC‐SD, or through regulating the plasticity of parental LLC cells to give arise to LLC‐SD.

Taken together, this study has provided convincing evidence that *Nr5a2* exerts its novel regulatory activity on lung CSCs and on driving lung tumorigenesis and progression through transcriptional activation of Nanog. We are undertaking studies to verify the regulatory mechanism reported here in other human lung CSCs and in new clinical lung cancer cohorts. Further investigations are necessary to corroborate these preliminary observations which have significant implications for improving the diagnosis, prognosis, and developing new targeted therapies for lung cancer.

## CONFLICTS OF INTEREST

The authors have no conflict of interests to declare.
