# Supplementary figures and images for: Nr5a2 promotes cancer stem cell properties and tumorigenesis in nonsmall cell lung cancer by regulating Nanog
Source: Cancer Med. 2019 Feb 10;8(3):1232–45. doi: 10.1002/cam4.1992 (PMC6434341; doi:10.1002/cam4.1992)

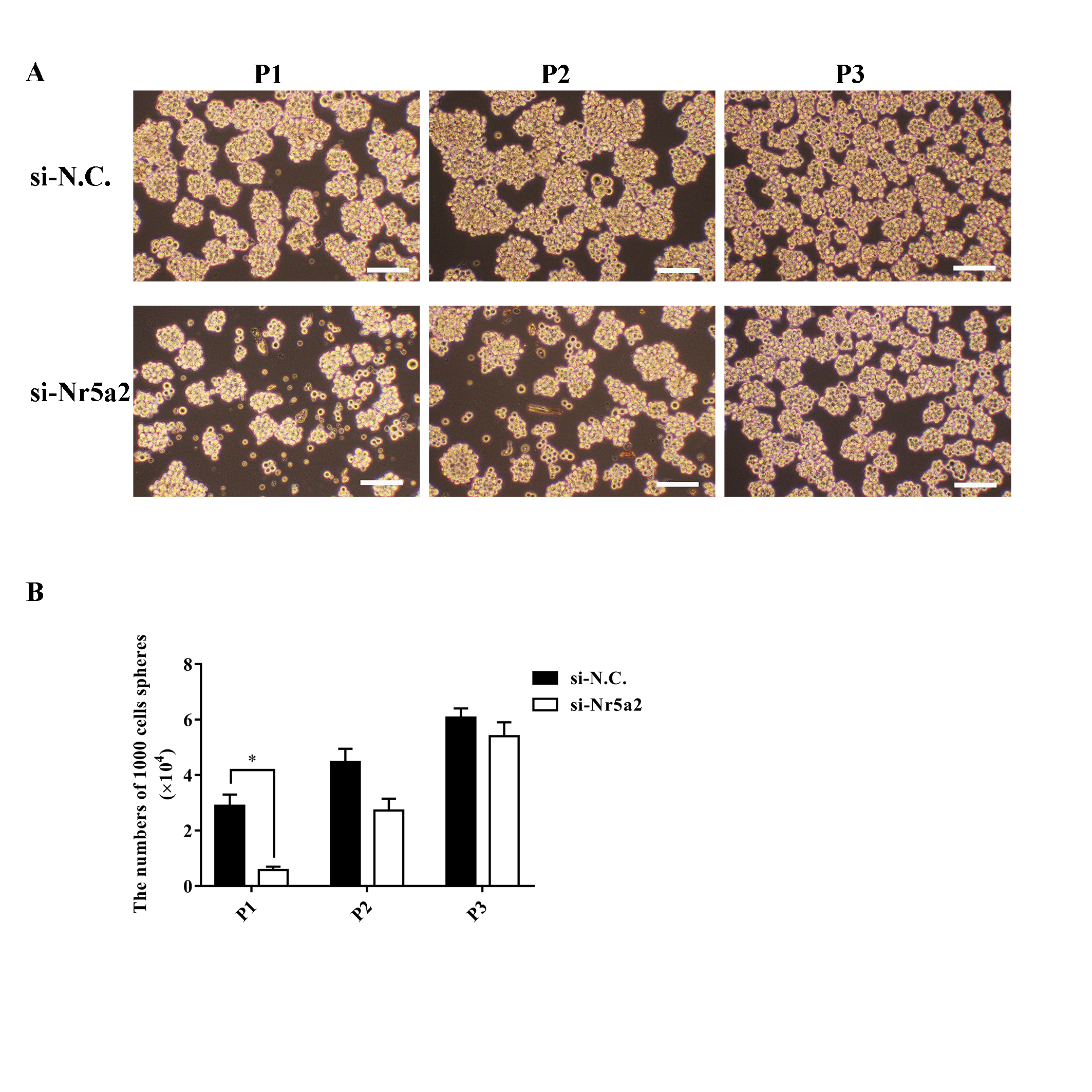

Supplement: Supplementary file 1 [file CAM4-8-1232-s001.tif]

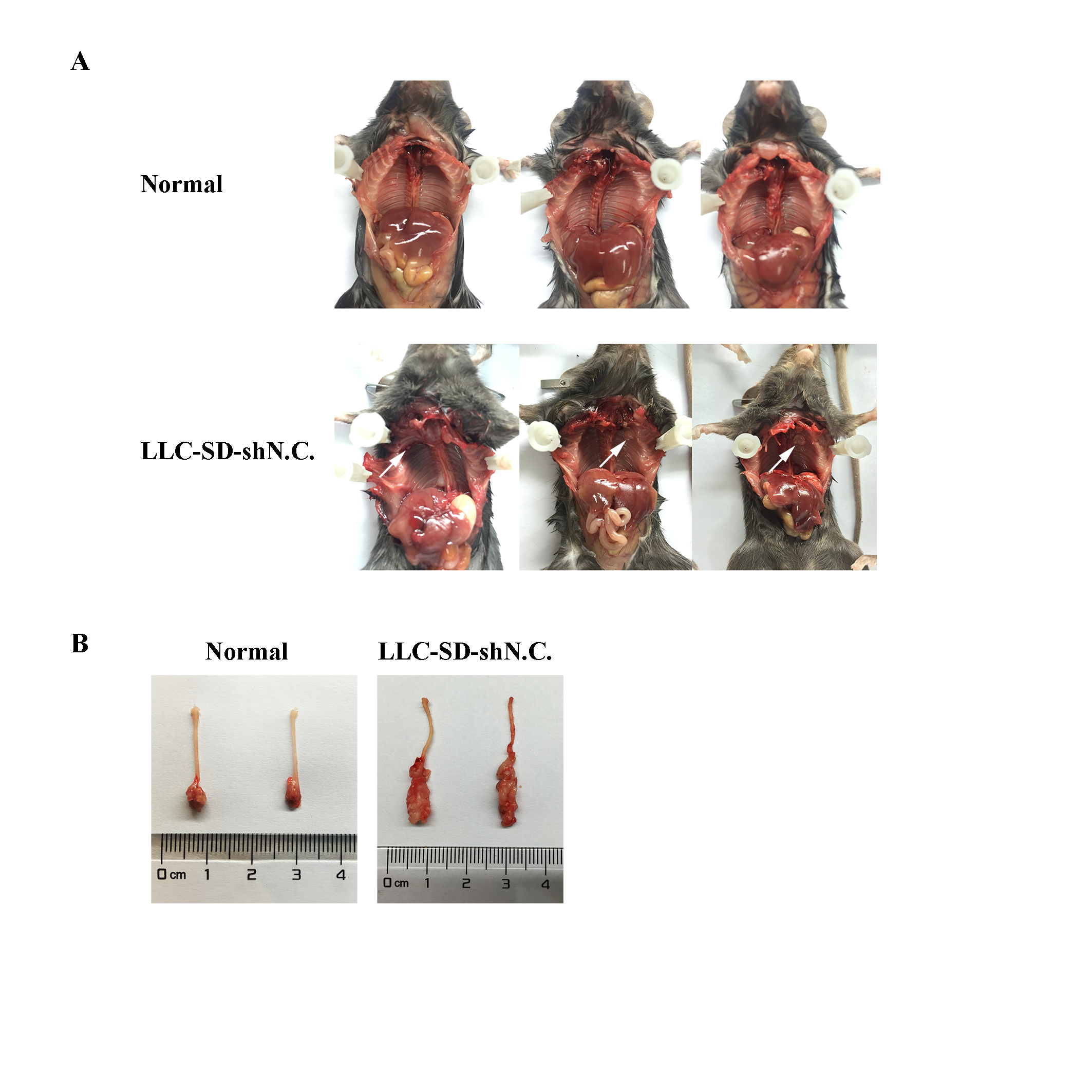

Supplement: Supplementary file 2 [file CAM4-8-1232-s002.tif]
